# The Relationship Between Phenological Characteristics and Life Forms Within Temperate Semi-Natural Grassland Ecosystems in the Central Himalaya Region of India

**DOI:** 10.3390/plants14060835

**Published:** 2025-03-07

**Authors:** Archana Fartyal, Ravi Kant Chaturvedi, Surendra Singh Bargali, Kiran Bargali

**Affiliations:** 1 Department of Botany, D.S.B Campus, Kumaun University, Nainital 263002, Uttarakhand, India; archanafartyal2008@gmail.com (A.F.); kiranbargali@yahoo.co.in (K.B.); 2 Yunnan Key Laboratory for Conservation of Tropical Rainforests & Asian Elephant, Center for Integrative Conservation, Xishuangbanna Tropical Botanical Garden, Chinese Academy of Sciences, Menglun, Mengla 666303, China

**Keywords:** biological spectrum, climatic condition, degraded land, growth form, phenology

## Abstract

The seasonal phenological segregation observed among various species within a plant community can be interpreted as a form of niche differentiation that facilitates the coexistence of these species. In the present study, life forms and phenological attributes of dominant plant species in temperate semi-natural grasslands of Central Himalaya, India, were assessed between January 2022 and December 2022. This study was carried out in three sites in different forest zones, viz. oak, cypress and pine. In each site, plots measuring 0.5 hectares were established and phenological assessments were conducted within each of these plots. A total of 50, 36, and 49 herbaceous species were identified in the grasslands of oak, cypress and pine zones, respectively, with these species categorized into five distinct life form classes. In the grasslands of both oak and pine zones, hemicryptophytes emerged as the predominant life form, whereas in the cypress zone grasslands, it was found that chamaephytes take precedence. The differences observed in the classifications of life forms can be ascribed to the geographical distribution and the biotic interactions present in these sites. The three grasslands exhibit comparable climatic conditions and day lengths, resulting in no significant variations in soil temperature, light intensity or overall climatic factors. The majority of species commenced their flowering phase during the monsoon season, attributed to the favorable conditions characterized by warm, humid weather and adequate soil moisture. Various phenological events, including germination, growth, and senescence, are significantly affected by weather and climate, and their timing subsequently influences ecosystem processes in a reciprocal manner. This study provides valuable foundational data for ecological and environmental research, aiding in the comparison and distinction of plant compositions across the Himalayas and its ecosystems.

## 1. Introduction

The timing of plant life stages and life forms are essential aspects of plant strategies because they influence the ability of plants to acquire variable resources. The study of plant life cycle timing, known as plant phenology, is linked to various ecosystem processes and functions [1]. Consequently, it maintains the capacity of any plant species to utilize resources or react to regulators that change throughout the year [2]. Life forms create a variety of physical traits related to gaining access to resources that are not evenly distributed above and below ground [3,4]. Phenology is mostly determined by the habitat and local climate (temperature and precipitation) in grassland ecosystems [5]; however it is also impacted by a variety of biotic and abiotic factors [6]. Several aspects of ecosystem structure and function, including leaf area, photosynthesis, carbon cycling, species diversity/composition, and conflict, are strongly influenced by the beginning, senescence, and length of growing seasons [7]. The extended duration of the growing season in temperate habitats due to climate change has dramatically altered plant phenology [8,9]. These alterations seem to occur almost universally among plant species and geographical levels (from plant species to landscape, region, and continental size) [9,10].

Determination of the variation in species lifecycles is facilitated by identifying the phenological attributes, which include life forms, growth forms and other phenotypic aspects. These qualities reflect the current ecological circumstances, microclimate, and evolutionary processes [11,12]. As a result, this may be applied to evaluate the ecosystem’s ecological health [13,14]. An ecological habitat’s phytoclimatic vegetation type is determined by the divergence from Raunkiaer’s normal spectrum, which displays a phanerophytic community [12]. The dominating life forms that define the phytoclimate of a particular ecosystem can be identified by comparing Raunkiaer’s normal spectrum with the biological spectrum of life forms [15]. Thus, in addition to plant composition and ecological diversity surveys, the study of the biological spectrum is a crucial component of ecological research and the characterization of vegetation ranks [16]. The distribution of various living forms within a region’s flora may be expressed using the biological spectrum [17]. The term “life-form spectrum” refers to all species that have adapted to a given environment [18]. It can serve as an indicator to gauge the state of a certain area since it depicts the ecological processes that exist now [11,19]. In ecological and biogeographical research, the biological spectrum is often employed for investigating vegetation types and plant communities [12,20], and it is helpful for comprehending plant survival strategies in an array of temperatures, climatic conditions and habitats [17]. The biological spectrum, growth forms, life forms, and floral diversity of India have all been studied by several authors to date from different locations [18,21,22,23,24]; however, despite the ecological significance of these grasslands, the phenology and life form classifications of plant species in this region are not well studied, particularly in relation to different life forms. By using Raunkiaer’s life form concept to categorize plants, we may study how various life forms endure and procreate in these grassland settings, where year-round variations in environmental parameters like soil temperature, moisture content, and light intensity occur.

Plant phenology is a significant aspect of plant ecology, emphasizing its importance across individual organisms to entire ecosystems. The precise timing of the change between vegetative and reproductive phases, which coincides with flowering, is critical to the best establishment of seeds for both individuals and populations [25]. As the phenological development of species in plant communities determines the pace at which herbage accumulates, a precise understanding of this process is critical to the management of herbage resources [26]. The study of plant phenology in grassland communities is important as recent climate change has emerged as a strong influence on the abundance, distribution, productivity and phenology of grassland communities [27]. Herbaceous plants in grasslands are especially sensitive to increasing temperatures and altered precipitation patterns because most of them are shallow-rooted, short-lived, and exhibit large and rapid responses to climate fluctuations, therefore providing insights into how climate change impacts plant life cycles, with variations in phenological events [8]. The phenology of individuals plays a key role in determining how ecosystems are structured and how they function, and is a key factor in the fitness and reproductive success of individual species, as well as in competitive interactions within and among species thereby, driving species distribution and community assemblages [28]. It is crucial to comprehend the phenology of these species in order to map plant survival strategies and evaluate their contribution to the grassland’s overall ecological function [8,12]. This study sheds light on the seasonal dynamics of plant development by recording the phenology of different life forms recorded in these habitats. Furthermore, the timing of actions like controlled fires or mowing to preserve species variety and habitat quality may be planned with the use of phenological data. By understanding the phenological shifts, scientist may anticipate ecosystem responses, direct conservation endeavors and manage land use to maintain and preserve biodiversity and productivity [29,30]. Gaining insight into the growth forms and phenological patterns of species present in grassland communities can assist useful in the optimization of grazing operations, resulting in lower economic losses and higher output [16]. Stakeholders can also modify their actions to preserve or improve ecosystem services by forecasting how climate change would affect grassland effectiveness. In the end, these studies help create plans and regulations that guarantee the protection and sustainable use of grassland ecosystems, which benefits both local populations and the environment at large [31].

The grasslands in India are developed as secondary successional communities in the denuded forestlands [32]. They are maintained mainly because of various biotic pressures caused by human being and animals [33]. The grasslands within the study area predominantly consist of unsown wild herbaceous communities, commonly classified as semi-natural grasslands. These ecosystems are characterized by their natural plant communities, which are primarily composed of various prostrate grasses. The maintenance of these grasslands is facilitated through grazing practices and the management of woody vegetation removal. Although semi-natural grasslands serve as a fundamental resource for livestock production in the Central Himalayan region of India, the understanding of these ecosystems remains fragmented and insufficient. This lack of comprehensive knowledge stands in stark contrast to the more extensive information available regarding grasslands and pastures in temperate and subtropical areas, primarily due to the scarcity of data concerning their ecological attributes. The objective of this study was to understand the capacity of grassland species to shift their morpho-phenological traits with respect to climatic conditions and to assess whether the phenology of grassland species differs among forest zones of a temperate region. Understanding grassland phenological responses may be particularly useful for predicting the species strategies and the functional parameters of communities within grassland ecosystems.

## 2. Results

### 2.1. Site Characteristics

**Figure 1:**
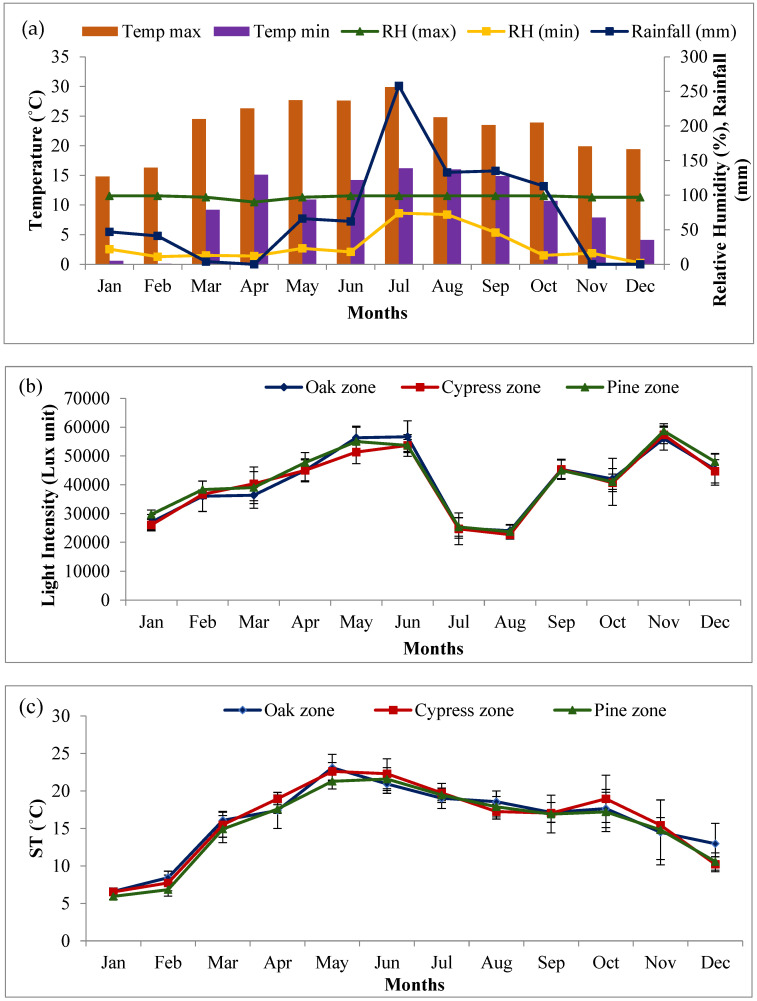
Monthly variation in climatic conditions (**a**), light intensity (**b**) and soil temperature (**c**) during the study period.

During the study period, between January 2022 and December 2022, maximum precipitation was recorded during the monsoon season (from mid June to mid October). During the rainy season, the study area had a cumulative rainfall of 767 mm, with 33.63% (258 mm) occurring in the month of July (Figure 1a).The minimum mean monthly relative humidity fluctuated between 2% (January) and 74% (July), while the maximum mean monthly relative humidity fluctuated from 90% (April) to 99% (rainy season). The highest mean maximum temperature was recorded during the summer season in the month of July (29.9 °C), while the lowest mean minimum temperature, 0.1 °C, was recorded during the winter season in the month of February.

Among the grassland sites, maximum light intensity was recorded for the pine zone grassland (58,667 Lux unit) in the month of November while minimum light intensity (22,667 Lux unit) was recorded for the cypress zone grassland in the month of August (Figure 1b). The light intensity ranged from 24,000 to 56,000 Lux unit in the oak zone grassland, from 22,667 to 57,333 Lux unit in the cypress zone and from 23,667 to 58,667 Lux unit in the pine zone grassland. The soil temperature during the study period showed insignificant differences (*p* > 0.05) between the grassland sites and fluctuated between 5.9 °C (January) in the pine zone grassland and 23.1 °C (May) in the oak zone grassland (Figure 1c). ANOVA showed that both the light intensity and soil temperature did not differ significantly among the grassland sites. However, a significant difference at *p* < 0.05 was recorded in both these variables due to the monthly variation.

### 2.2. Floristic Composition and Life Forms

**Figure 2:**
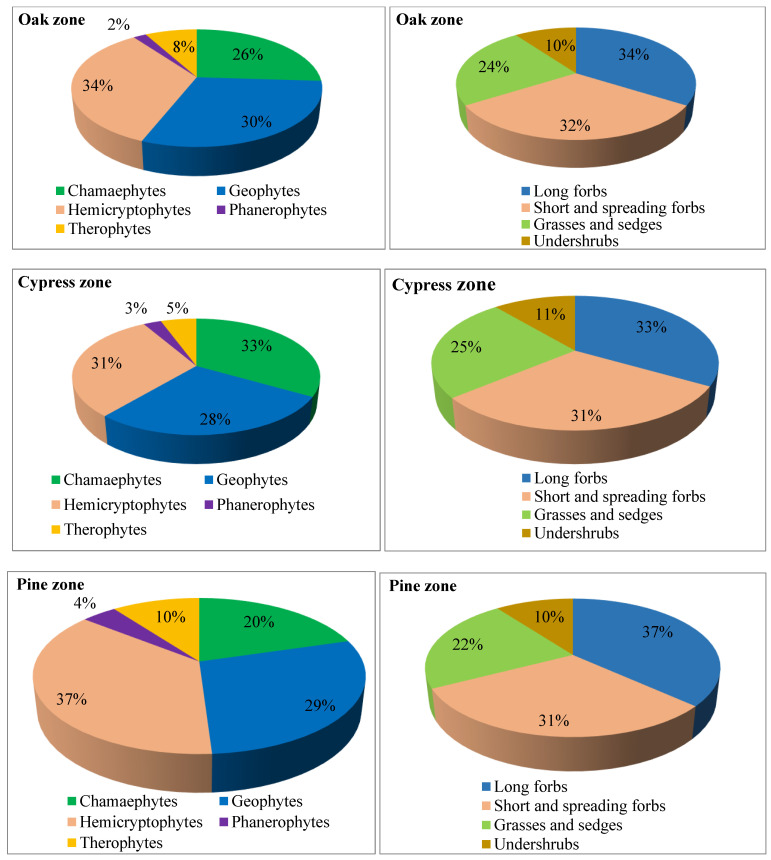
Proportional distributions of species in different categories of life form and growth form.

A total of 50, 36 and 49 species were recorded from the oak, cypress and pine zone grasslands, respectively (Table 1). Out of the 50 plant species recorded in the oak zone grassland, 26% were chamaephytes, 30% were geophytes, 34% were hemicryptophytes, 2% were phanerophytes and 8% were therophytes. In the cypress and pine zone grasslands, about 33.33% and 20.41% were chamaephytes, 27.78% and 28.57% were geophytes, 30.56% and 36.73% were hemicryptophytes, 2.78% and 4.08% were phanerophytes and 5.56% and 10.20% were therophytes, respectively (Figure 2).

On the basis of the growth forms, long forbs and short and spreading forbs made up about 34% and 32% in oak zone grasslands, 33.33% and 30.56% in cypress zone grasslands and 36.73% and 30.61% in pine zone grasslands, respectively. The proportion of grasses and sedges were 24%, 25% and 22.45% in oak, cypress and pine zone grasslands, respectively. However, undershrubs showed almost similar distributions in all the three sites (Figure 2). According to the plant growth classification scheme by IUCN, approximately 43% (oak zone), 42% (cypress zone) and 35% (pine zone) of species contributed to forbs or the herb growth form; 25% (both in oak and cypress zone) and 23% were (pine zone) identified as graminoids; 4%, 5% and 10% species were observed to be annuals in oak, cypress and pine zones, respectively. Of the total species, geophytes contributed 20%, 17% and 22% of the total, while small shrubs accounted for 4%, 5% and 10% in oak, cypress and pine zone grasslands, respectively, while all other growth forms were absent from all the grassland sites.

### 2.3. Community Level Phenology

**Figure 3:**
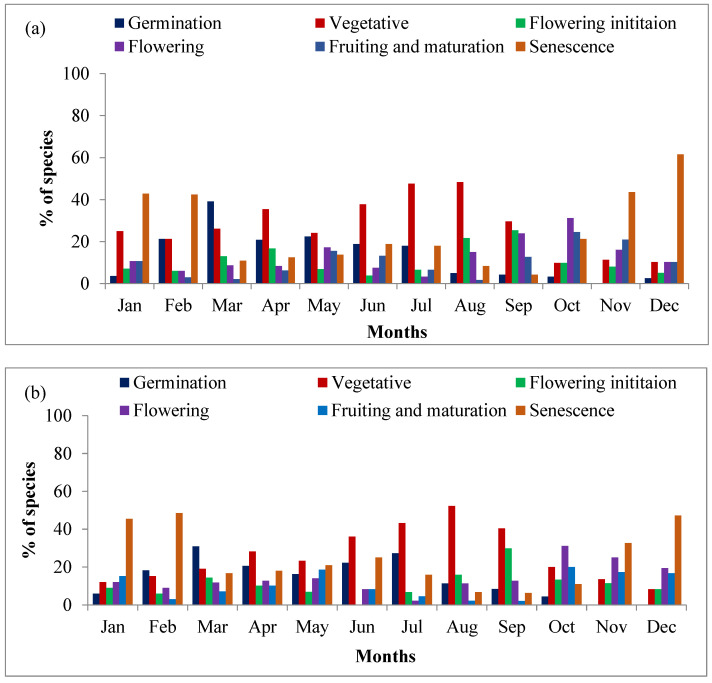

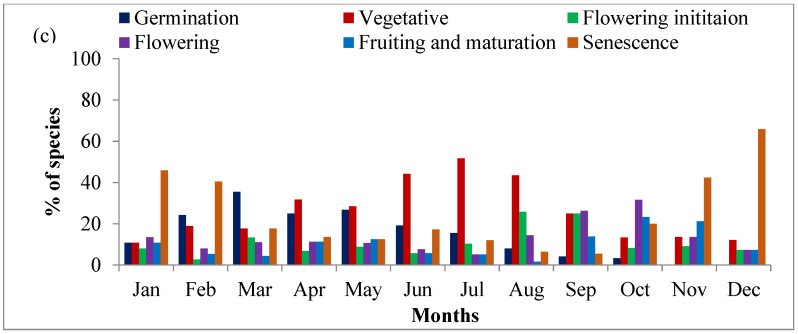
Percentage of the species under different phenophases in different months—(**a**) oak zone grassland, (**b**) cypress zone grassland and (**c**) pine zone grassland.

Of the total plant species examined for phenophases in all the three zones, a substantial portion of plant species begins to germinate with the advent of the spring season (March), which includes species like *Ageratina adenophora*, *Anaphalis contorta*, *Artemisia annua*, *Barleria* cristata, etc. In the oak and cypress zone grassland, approximately 39.13% and 30.95% of species, respectively, initiated germination in March, with the arrival of the spring season. The germination percentage decreased thereafter and again increased with the advent of rain showers in the month of June and July. However, in the pine zone grassland, 30.95% of the species initiated growth while 22.22% of species started germinating with the first sustained rains in June. In most of the grass species, seedlings of annuals and perennial species initiated growth at the end of the May with the advent of rain. By and large, the germination of species continued with the advent of rains during late June and early July. The germination and sprouting of seeds and perennating buds were followed by vigorous vegetative growth, due to which the bare area became lush green in color. The vegetative phase of most of the species peaked in July and August, after which they started flowering. Most of the species started flowering (floral induction) in the month of September, while the peak of flowering was recorded in the month of October (first week of October); along with this, fruiting was also observed in number of species in the month of October. This pattern was consistent across all the zones, where species showed a synchrony in entering their reproductive phase after the peak vegetative growth in the rainy season. Some of the rainy season annuals such as *Arenaria serpyllifolia*, *Artemisia annua*, *Arthraxon lanceolatus*, *Cynoglossum furcatum*, *Gonostegia hirta*, etc., started flowering by the end of August and early September, while in most of the perennial grasses, flowering started during September and fruiting was recorded by the end of November. As the temperature dropped, most of the species underwent senescence, which marked the end of their life cycle. This phase was especially evident in December, when the majority of species across all zones entered senescence. The percentage of species under different phenophases in different months is demonstrated in Figure 3.

### 2.4. Phenological Attributes of Species

#### 2.4.1. Vegetative Phase

**Figure 4:**
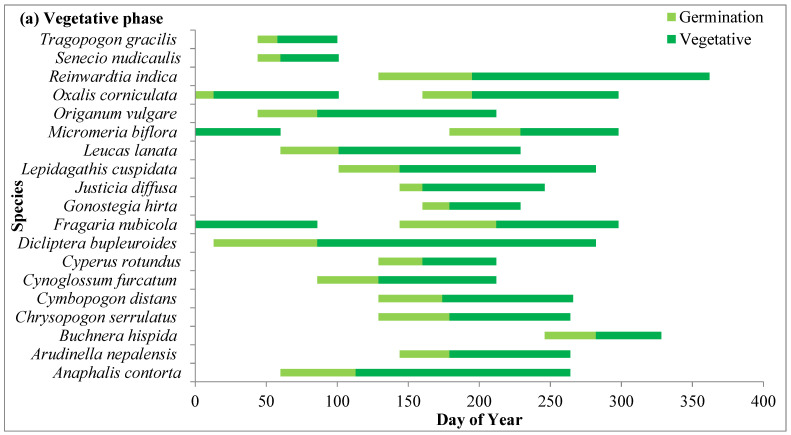

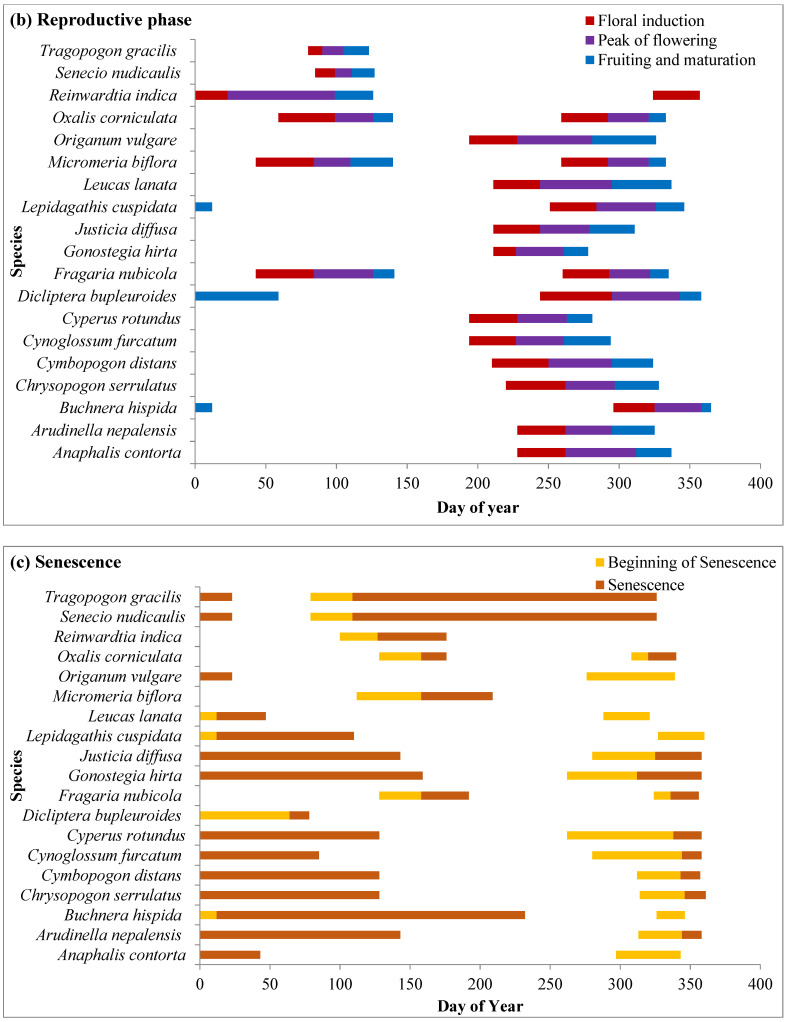
Phenophases for dominant plant species representing (**a**) vegetative, (**b**) reproductive and (**c**) senescence phases of dominant species in oak zone grassland.

**Figure 5:**
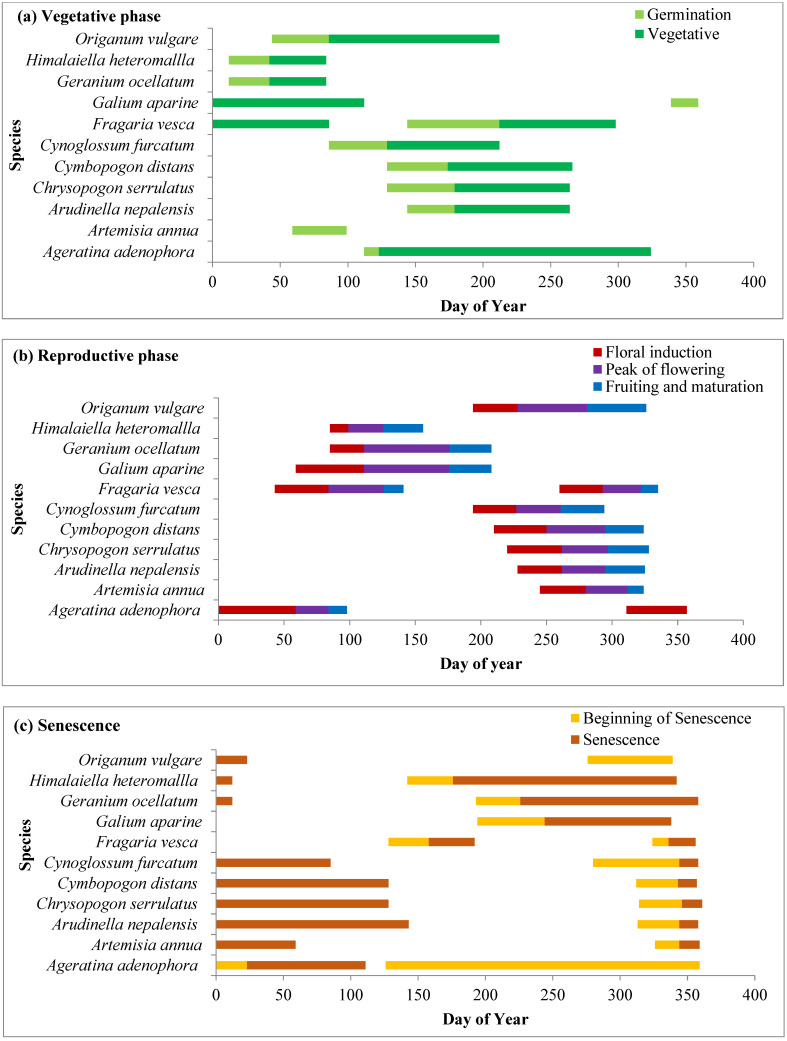
Phenophases for dominant plant species representing (**a**) vegetative, (**b**) reproductive and (**c**) senescence phases of dominant species in cypress zone grassland.

**Figure 6:**
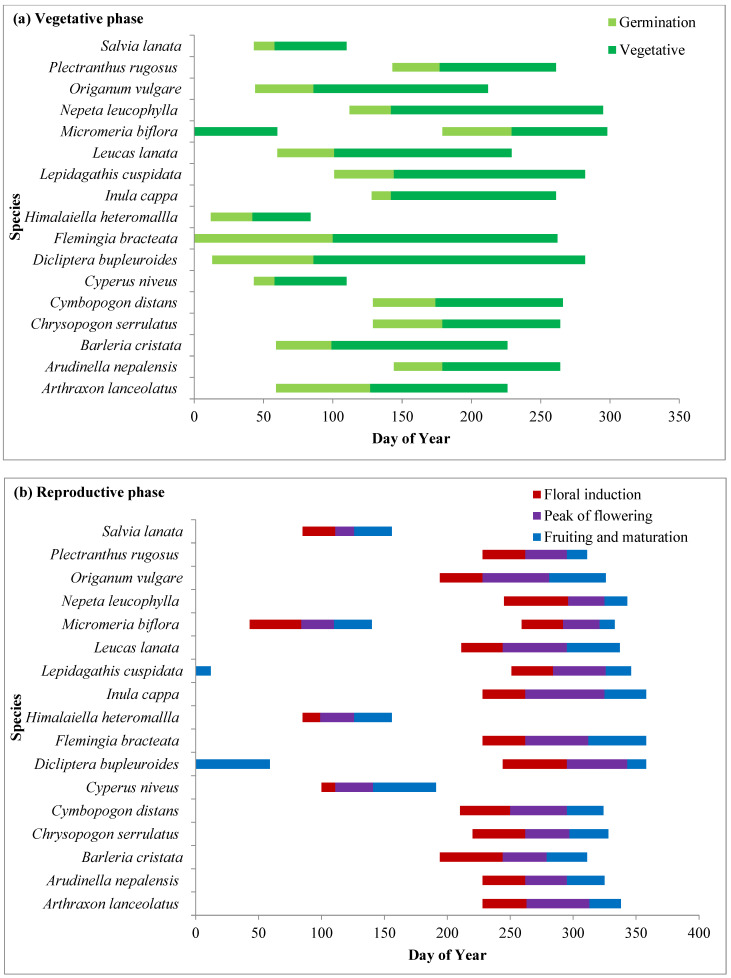

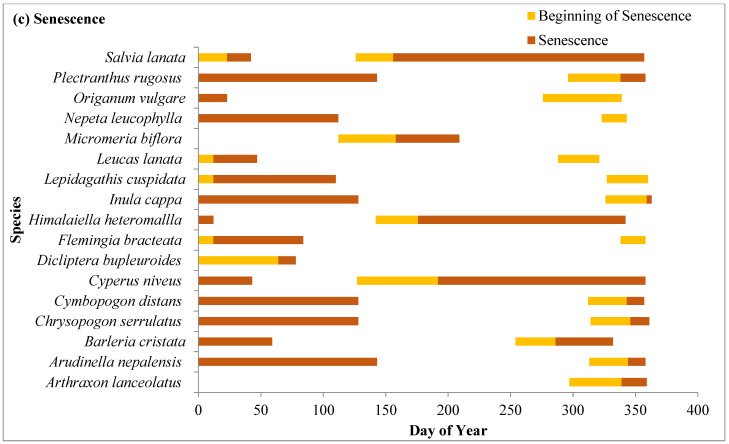
Phenophases for dominant plant species representing (**a**) vegetative, (**b**) reproductive and (**c**) senescence phases of dominant species in pine zone grassland.

Phenophases of dominant species in different grassland zones are represented in Figure 4, Figure 5 and Figure 6. In the present study, species including *Ageratina adenophora*, *Artemesia annua, Anaphalis contorta*, *Cynoglossum furcatum*, *Lepidogathis contorta*, *Leucas lanata*, *Origanum vulgare*, *Senecio nudicaulis* and *Tragopogon gracilis* germinated with the advent of the spring season in the month of March. Two species, viz. *Senecio nudicaulis* and *Tragopogon gracilis*, showed short growth cycles and completed their life cycle within 3–4 months before the rainy season. Grass species such as *Arudinella nepalensis*, *Chrysopogon serrulatus*, and *Cymbopogon distans* germinated rapidly with the beginning of the rainy season in the month of June and other forbs such as *Gonostegia hirta* and *Micromeria biflora* also started germinating with them. *C. niveus*, which is early-growing sedge, and forbs such as *Flemingia bracteata, Geranium ocellatum*, *Himalaiella heteromalla*, and *Salvia lanata* started germination during the winter. Germination of other species took place during the pre-monsoon season (April to May). After germination, the species underwent rapid vegetative growth during the rainy season (July to September) and their growth peaked in July and August.

#### 2.4.2. Reproductive Phase

The reproductive phase of *Fragaria vesca*, *Galium aparine* and *Geranium ocellatum* started in the month of March while that of *Himalaiella heteromalla* started in April. *Himalaiella heteromalla* completes its life cycle within 4–5 months and starts senescence in the month of June. Three species, *Cynoglossum furcatum, Justicia diffusa* and *Origanum vulgare*, started flowering in the last week of July. *A. adenophora* started flowering earlier in winter and ended in the early summer season after day of year (DOY) 90 while *Artemisia annua* started flowering during the monsoon season and lasted upto after the monsoon season (late October). Flowering initiation peaked in the month of September as a significant proportion of grass species (*Arudinella nepalensis*, *Chrysopogon serrulatus*, *Cymbopogon distans*) and some forbs initiated flowering in this month. Flowering in species increased from July to October and maximum flowering was recorded in the first week of October. In most of the species, fruiting started from the last week of October, and the peak was recorded in November, after which it declined subsequently during winter. However, in *Gonostegia hirta*, fruiting started in the month of September.

#### 2.4.3. Senescence Phase

Senescence in grass species and forbs occurs during the winter season, ensuring that they complete their life cycle before the extreme winter conditions. *Artemisia annua* and *Fragaris vesca* showed complete senescence of aboveground parts in the winter, while in other species, senescence ended either towards the end of winter or in the middle of the summer season of the following year.

### 2.5. Relationship Between Phenological Attributes and Growth Forms

The germination and growth initiation in long forbs (47.37%) and grasses (60%) peaked in March. However, among the short forbs and undershrubs, germination peaked in July (31.03%) and April (42.86%), respectively. Vegetative growth in short forbs and grasses peaked in August, while in undershrubs and tall forbs, it peaked in June and July, respectively. Flowering in long and short forbs occurred earlier, peaking in September as compared to grasses, for which flowering peaked in October. Except for short forbs, all growth forms showed seed maturation and senescence during the post-monsoon or winter season, despite differences in phenophases. However, in short forbs, fruiting and seed maturation peaked in the month of May due to the presence of plants with a short growth cycle.

Among the life forms, out of the total therophytes and phanerophytes, 50% started germinating in the month of February and March, respectively. The vegetative growth of both these life forms peaked in the month of July and they entered the reproductive phase during the rainy season in the month of September. Hemicryptophytes and chamaephytes began germination with the advent of the spring season, and their germination peaked in March. The vegetative growth of hemicryptophytes peaked in the month of July, while chamaephytes attained maximum growth in the month of June. Most of the hemicryptophytes and chamaephytes underwent the reproductive phase during the rainy season and experienced maximum senescence during the winter. The germination of geophytes peaked in the month of July, achieved peaked growth in the month of August, underwent the reproductive phase in September and underwent senescence during the winter season.

### 2.6. Relationship Between Phenological Attributes and Climatic Conditions

**Figure 7:**
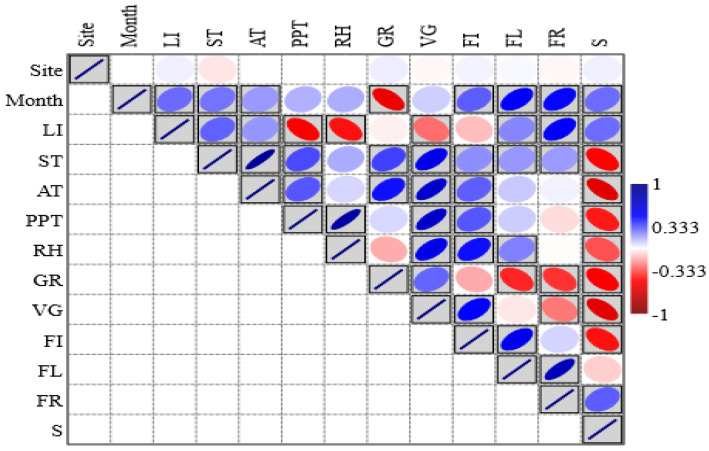
Correlation matrix among climatic parameters and phenophases (where LI = light intensity; ST = soil temperature; AT = average temperature; PPT = precipitation; RH = relative humidity; GR = germination; VG = vegetative; FI = flowering induction; FL = flowering; FR = fruiting and maturation; S = senescence).

**Figure 8:**
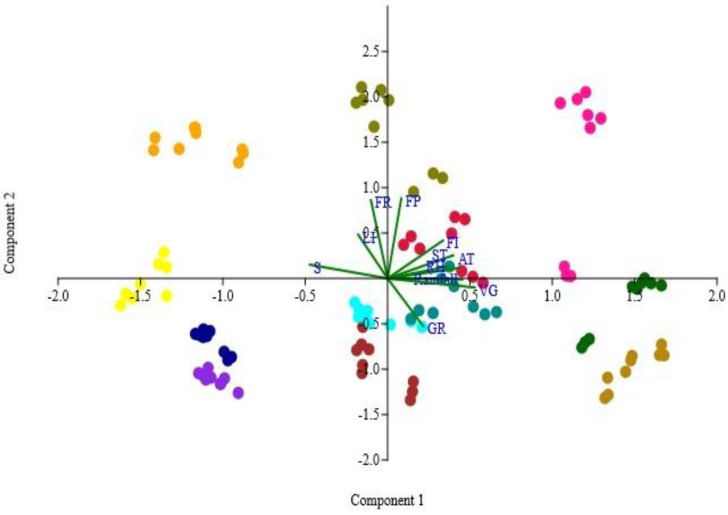
Principal component analysis (PCA) correlation plot of climatic parameters and phenophases of species in different months (January = blue; February= violet; March = brown; April = aqua; May = crimson; June = darkcyan; July = golden; August = green; September = pink; October = olive; November = orange; December = Yellow; LI = light intensity; ST= soil temperature; AT = average temperature; RH = relative humidity; GR = germination; VG = vegetative; FI = flowering induction; FL = flowering; FR = fruiting and maturation; S = senescence.

Pearson’s correlation between various climatic factors and phenophases revealed significant positive relationship for germination (r = 0.373, *p* < 0.05), vegetative growth phase (r = 0.584, *p* < 0.05), floral induction (r = 0.220, *p* <0.05), flowering peak (r = 0.203, *p* < 0.05), and fruiting (r = 0.197, *p* < 0.05) with soil temperature. However, a significant negative correlation was observed for senescence at the *p* < 0.05 level of significance, indicating that senescence increased with decreasing soil temperature. Light intensity showed a negative correlation with both germination and vegetative growth, with a significant relationship only for germination. In the reproductive phase, peak flowering and fruiting exhibited a significant positive correlation with light intensity (*p* < 0.05). The average temperature (AT) of the study sites also showed a positive correlation with germination (r = 0.469), vegetative growth (r = 0.694), floral induction (r = 0.315) and the flowering (r = 0.105) and fruiting (r = 0.026) phenophases; however, the relationship was significant only for the germination, vegetative growth and flower initiation phases at *p* < 0.05. The amount of precipitation during the study period was positively correlated with germination, vegetative growth, flower initiation, and flowering peak, while a negative correlation was observed for the senescence phase. However, the relationship was insignificant for germination and flowering (Figure 7). Principal component analysis identified 11 principal components, in which PC1 and PC2 contributed 37.70% (eigen value = 4.14) and 22.69% (eigen value = 2.49) to the variance, respectively (Figure 8). PC1 is an environmental situation that is favorable for plant development, as indicated by the significant positive loading values for vegetative growth (0.91) and precipitation (0.77) and the negative loading value for senescence (−0.89). The positive relationship between vegetative growth and rainfall indicates that better moisture availability is associated with more vigorous vegetative growth, while the inverse relation of this component with senescence means that due to the reduction in these growth conditions, plants reach senescence. On the other hand, PC2 exhibits positive loadings for soil temperature (0.68), flowering peak (0.91), and floral induction (0.89), indicating that this component depicts the impact of temperature on the reproductive stages of species. In PC3, positive loadings of AT (average temperature) and rainfall indicate that months with higher temperatures and more rainfall are associated with higher values of this component, while the negative loading of RH (relative humidity) suggests that months with lower humidity and higher temperature and moisture availability correspond to higher values of PC3. Temperature and moisture availability are important factors that influence plant reproductive success, and this component most likely reflects the climatic conditions that encourage fruiting, although humidity may mitigate these effects.

## 3. Discussion

Phenophases in temperate regions are highly seasonal and vary across a significant portion of the year due to temporal fluctuation in both abiotic and biotic environments [36]. Temperature and precipitation are two major climatic factors that influence the beginning of the vegetative season in temperate locations [37,38,39]. In the present study, grasslands experienced similar geographical, climatic, and day length conditions and did not show any significant differences in soil temperature, light intensity or climatic conditions. The life form spectrum showed the dominance of hemicryptophytes in oak and pine zone grassland sites, while chamaephytes were more prevalent in cypress zone grassland. Similar biological spectrums seen in several locations are indicative of similar climatic conditions [12]. The dominance of hemicryptophytes in this study was possibly due to the frigid and mountainous climate of the region. Typically, these plants withstand dry spells or periods of water scarcity by means of physiological, morphological, and anatomical modifications that minimize water loss [40]. According to Jayanthi and Jalal [41], hemicryptophytes are seasonal and occur more frequently in grasslands during the rainy season. The region experiences its highest rainfall from July to September, creating favorable soil and air temperature conditions that support the growth of these species, resulting in the greatest diversity during September. Shimwell [42] and Cain and Castro [43] suggested that the dominance of hemicryptophytes is indicative of temperate zones, while Surmal et al. [12] reported that chamaephytes and hemicryptophytes are restricted to colder climates, and that a rise in the number of phanerophytes and therophytes indicates that the community has a warmer environment [12]. Horbach et al. [44] suggested that a high degree of phenological homogeneity within a particular life form indicates a strong temporal and spatial co-variation in resource utilization. In all the grassland sites, out of the total species phanerophytes contributed 2, 3 and 6% in oak, cypress and pine zone grasslands, respectively. The low number of phanerophytes in grasslands may be attributed to the periodic fire events, which prevent phanerophytes from growing quickly. However, hemicryptophtyes in grassland communities are well adapted to fire, which allows them to survive and regenerate after a fire. A complete absence or low proportion of phanerophytes was reported by Singh and Ambasht [45] in Varanasi grassland, Singh and Joshi [46] from a meadow near Pilani in Rajasthan, Singh and Yadava [47] in tropical grassland of Kurukshetra, and Rout and Barik [48] from Bagiiriposi, Odisha. The Raunkiaer normal spectrum and biological spectrum of some Indian grassland communities are given in Table 2.

There are notable seasonal variations that coincide with the shift in the species phenology within the community [60]. In all the sites, grasses start germinating with the advent of few rain showers in the end of May and most species showed peak development stages that corresponded with high levels of relative humidity, rainfall, and soil temperature during the warm, rainy monsoon months (August and September). The high moisture and temperature during the monsoon season is closely associated with the growth of many species, with germination and vegetative growth reaching their peak in July and August. This coordinated timing of germination, vegetative growth, blooming, and fruiting benefits not just the specific species but also the entire ecosystem by providing a consistent supply of several resources [61]. Forbs like *Anaphalis contorta*, *Cynoglossum furcatum*, *Leucas lanata*, *Origanum vulgare*, *Senecio nudicaulis*, *Tragopogon gracilis*, etc., start germinating earlier before the advent of the rainy season during spring or the pre-monsoon season, which leads these species to reduce competition for light, nutrients and other resources. In the present study, most of the species showed signs of germination during the spring season to benefit from the ideal climatic conditions, such as the rising temperatures and longer day lengths, which are essential for optimal plant establishment and development [62]. Temperature and moisture, therefore, appear to have an impact on these species’ ability to germinate [63,64]. For each combination of environmental factors, there exists a group of species with similar traits that tend to occur together more often than expected by chance. Currie and Sala [65] also observed that temperature has little impact on plant development, whereas precipitation has a strong significant correlation with the timing of vegetative growth and senescence. Plant species undergo early senescence when there is insufficient precipitation, whereas increased precipitation causes delayed senescence [66]. Species such as *Tragopogon gracilis, Senecio nudicaulis,* and *Himalaiella heteromalla* complete their life cycle within the short period of time and thrive through their dormant, perennating buds. As a result, these plants that thrive in different ecosystems tend to bloom during the spring season [67]. Kala [67] investigated the phenology of sub-alpine as well as alpine plants in Western Himalaya and reported seed dehiscence during September and October, and the best conditions for growth during April, indicating considerable diversity in regenerative traits with relative uniformity in growth and reproduction-related traits within the plant community. Pangtey et al. [68] also reported that plant species in high-altitude grassland ecosystemcompletes their development cycles within a short period under favorable environmental circumstances. Pearson’s correlation showed that germination was negatively correlated with temperature as most of the plant species showed the lowest germination during the winter season. Bhattacharya [69] suggested that low temperatures are one of the abiotic factors that negatively impact plant species’ germination and vegetative development.

The flowering phenophase describes a specific phase of a plant’s life cycle and includes all of the noticeable blossoming-related occurrences and transformations, such as bud development, growth, and maturity, flower opening, and final senescence (aging and withering) [70]. Few studies have described the relationship between precipitation and flowering phenology in temperate zone communities [71]. In the present study, in most of the species, flowering began during the monsoon season because the warm, humid weather, easy access to water, and nutrient-rich soil conditions owing to the high decomposition rate providedan ideal environment for the plants. A study carried out by Bowers and Dimmitt [72] demonstrated that several environmental factors other than temperature and photoperiod also play an important role in the timing of flowering. Additionally, Ahmad et al. [73] noted that in temperate climates, warm temperatures typically serve as a trigger for flowering. The fruiting peak was observed during the autumn season, and a similar finding was reported by Upadhaya et al. [63], which indicated that plant species are greatly influenced by the weather patterns both within and between years. Peaks in soil temperature and rainfall corresponded with the blooming time for most related species. Compared to species blooming and fruiting in the pre-monsoon season (March–April), most of the species showed blooming and fruiting during the monsoon season (September and October). Because grassland ecosystems are water-limited, precipitation plays a crucial role in shaping vegetation dynamics in these ecosystems, and the timing and amount of rainfall directly influence plant phenological patterns [73,74,75,76,77]. The PCA also confirmed that environmental factors such as temperature, light intensity, and precipitation interact with different stages of plant growth, including germination, vegetative development, floral induction, flowering, and fruiting. Higher rainfall, lower light intensity and less senescence are linked to robust vegetative growth, indicating that wetter conditions and low light intensity result in more vegetative development and promote longer-lasting plant development. PCA results also reflect that temperature is a critical factor in determining the timing and intensity of reproductive events where warmer sites experience earlier or more intense flowering. Additionally, the research emphasizes the significance of floral induction (FI) and germination (GR) as ecological processes that are strongly related to environmental factors, with germination probably being impacted by moisture availability and temperature. These findings also suggested that temperature plays a critical role in both the early and reproductive phases of plant development. Additionally, precipitation also significantly affects plant growth and development patterns. A study conducted by Manila-Fajardo et al. [78] explained that temperature had a minor impact on blooming whereas rainfall had a considerable impact; also, temperature and rainfall have little effect on fruiting. In this study, precipitation showed a negative relation with the senescence phase and a positive correlation with germination, vegetative development, and floral induction. This suggests that moisture availability is essential for maintaining the early and intermediate periods of plants, whereas shortage of water causes senescence. Several other studieshave also revealed that water availability is a critical factor in determining plant production and reproductive success [61,79]. Since precipitation and senescence are negatively correlated, plants may achieve senescence faster with decreasing rainfall. This highlights the significance of moisture in maintaining vegetative development and postponing the beginning of senescence [80]. Although this study observed no significant relationship between precipitation and germination or flowering, it suggests that other variables, such as soil nutrients or temperature, may also have an impact on these phases [31]. This intricacy suggests that plant phenology is influenced by a variety of environmental variables, some of which may be disrupted by climate change.

The present study reveals the phenological diversity for different phenophases among plant species growing in temperate grasslands. This study would be of great help in knowing the timing of different phenophases of the studied plants, which can be of interest to people of this region because the changes in the phenological patterns can result in adverse effects on insect pollinators as well as herbivores that depend on those species for food.

## 4. Materials and Methods

### 4.1. Study Area

**Figure 9:**
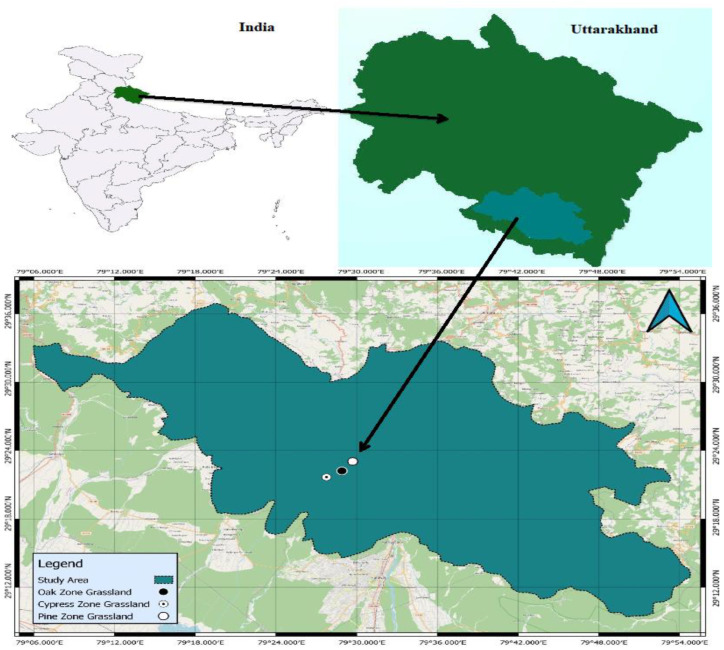
Map of the study area.

This study was conducted in the Nainital Forest Division, extending between 29°21′41″ and 29°22′56″ N latitude, and 79°27′45″ and 79°29′51″ E longitude in the Central Himalayan region, India (Table 3; Figure 9). The climate of the study area is of a temperate and monsoon type, with a mean maximum temperature of 29.9 °C in the summer and mean minimum temperature of 2 °C in the winter. The area receives approximately 859 mm of average annual rainfall, out of which 81% of rainfall was received during the rainy season (June to mid-October), which was governed by the southwest monsoon [81]. However, local convections also caused some precipitation during the pre-monsoon season. Geologically, the study area lies in the lesser Himalayan region and is dominated by the lithostratigraphic unit (a Krol series group of rocks), which mainly comprises sandstone, slate, dolomite and limestone. In the region, the tree layer is primarily dominated by *Quercus leucotrichophora* A. Camus, *Pinus roxburghii* Sarg., *Cupressus torulosa* D. Don, *Rhododendron arboreum* Sm., and *Cedrus deodara* (Roxb.) G. Don; the shrub layer is dominated by *Berberis asiatica* Roxb. Ex DC., *Rubus ellipticus* Sm., *Lantana camara* L., and *Himalrandia tetrasperma* (Wall. ex Roxb.) T. Yamaz. and the herb layer is dominated by *Cymbopogon distans* Nees ex Steud., *Chrysopogon serrulatus* Trin., *Arudinella nepalensis* Trin., and other plant species [32,70]. However, changes in vegetation are observed with changes in altitude, temperature, slope aspect and slope angle. The grasslands in this region are formed by the extensive degradation of forests [82,83] and maintained by grazing and fire. The soil of the study area is sandyloam to sandy clay loam in texture, and it is slightly acidic in nature (pH 5.9–6.4). A detailed description of soil physico-chemical properties of the study sites are given in Table 4.

### 4.2. Data Collection and Analysis

After a thorough reconnaissance survey, three semi-natural grasslands sites were selected in the oak (*Quercus leucotrichophora* A. Camus), cypress (*Cupressus torulosa* D. Don), and pine (*Pinus roxburghii* Sarg.) forest zones. According to Champion and Seth [84], the classification of the oak zone belongs to the lower western Himalayan temperate forest (Oak = 12/C1a), that of the cypress zone belongs to the Himalayan Moist temperate forest (Cypress = 12/E1) and that of the pine zone belongs to the upper or Himalayan Chir pine forest (Pine = 9/C1b). According to Joshi [83] and Rawat [85], grasslands below 3000 m of elevation were created by the massive degradation of forests approximately three to four decades ago. The selected forest zones are present near human settlements and are influenced by various anthropogenic disturbances such as cutting, lopping, collection of fuelwood, removal of litter, grazing by livestock and periodic fire events [86,87], which lead to the formation of large stretches of grasslands in these forest zones [83]. In addition, they symbolize a type of degraded system with several grazing tracks and fire signs which is subjected to increasing soil erosion.

In each forest zone, a 0.5 ha area of grassland was marked and a permanent plot of 20 m × 20 m was established. Within each permanent plot, 10 quadrats of 1 m × 1 m were placed randomly and 5 individuals of each selected species were tagged and visual phenological observations were recorded at 15-day intervals for one year (January to December 2022). Phenology was assessed through the identification and tracking of various phenophases that represent key stages of plant development throughout the growing season. We classified phenological phases into three main phases, i.e., the vegetative phase, reproductive phase and senescence phase. Further, the vegetative phase was divided into the germination stage, i.e., the first visible sign of a seedling sprouting from the soil surface, and the vegetative stage (the first appearance of leaves or shoots). Once a seedling was observed, it was tagged, and the date of emergence was recorded. The reproductive phase was separated into three stages, including floral induction (when floral primorida are visible in the plants inflorescence or terminal shoots), the peak of flowering (when the flower buds began to open fully (Enthesis), exposing the reproductive structures) and fruiting and maturation (when flowers were fully open (Anthesis), with the reproductive structures exposed). The last senescence phase was partitioned into the beginning of senescence (when leaves or flowers began to show visible signs of aging, such as discoloration, drying, or wilting) and the complete senescence stage (complete wilting, drying, and eventual loss of leaves, flowers, and stems) [70]. To normalize the timing of phenological events, the day of year (DOY) system was employed. For each phenophase event, the day of year was recorded, and it was calculated based on the calendar date of the observation, with day 1 corresponding to 1st January. For each 15-day interval, the phenological stage of each species was recorded as a specific DOY corresponding to the first observable occurrence of a phenophase within that period. In order to evaluate the overall data, phenophase calendars for species were developed and examined [88]. For the studied species, the frequency of occurrence of different phenophases in each month was calculated. The phenophase of a species was deemed to have begun when at least 10% of the individuals of that species had become apparent, and when less than 10% of the individuals remained in the phase, the phenophase was considered to have been complete for that species [70].

The meteorological data (relative humidity, temperature and precipitation) from January 2022 to December 2022 were collected from a nearby meteorological station (Aryabhatta Research Institute of Observational Sciences (ARIES), Nainital) located at 29.3592° N latitude and 79.4580° E longitude, and the soil temperature and light intensity of the study sites were recorded with the help of a soil thermometer and lux meter, respectively, at monthly intervals. The soil temperature was measured by inserting a soil thermometer into the soil upto a depth of 15 cm and leaving it there until the temperature stabilized, after which readings were recorded. The light intensity of the study sites was recorded using a lux meter during clear daylight conditions, by placing the sensor of the lux meter in a suitable location without any cloud cover and other obstructions. The sensor of the meter was placed horizontally to capture the light accurately, and was allowed to stabilize. Once the reading was stable, the light intensity was recorded in Lux (lx) units, as displayed on the meter.

The species were identified in the taxonomy laboratory of D.S.B. Campus, Nainital, with the help of available web resources and previous studies, such as those on Flora of Nainital district [34] and Flora Nainitalensis [35]. All the species were classified into different growth forms and life forms following Joshi [83]: (1) undershrubs, (2) tall forbs (species with heights of more than 30 cm), (3) short and cushion-forming forbs (species with less than 30 cm heights),and (4) grasses and sedges. The listed species were further categorized into a suitable Raunkiaer’s life form class based on the position of the perennating buds of plant species as phanerophytes (Ph), hemicryptophtyes (He), chamaephytes (Ch), geophytes (Ge), and therophytes (Th), respectively. Thereafter, it was compared with the Raunkiaer [3] normal biological spectrum and other previous studies. The plant species were also classified in different plant growth forms by IUCN (https://nc.iucnredlist.org/redlist/content/attachment_files/nov_2013_plant_growth_forms_classification_scheme.pdf (accessed on 2 January 2025)).

### 4.3. Statistical Analysis

One-way ANOVA was performed to determine the statistical difference in climatic data, light intensity, soil temperature and various phenophases due to the sites and months using SPSS software (version 22). Pearson’s correlation was computed to establish the relationship between the climatic condition and phenophases of species. Principal component analysis was employed to examine the relationship between environmental conditions and different phenophases of species across different months using PAST software (Version 4.03). The dataset includes the number of species observed in specific phenophases (germination, floral induction, flowering, etc.) for each month, along with corresponding climatic variables (temperature, precipitation, light intensity, etc.).

## 5. Conclusions

The inherent ecological abundance of the environment and the physiognomy of the vegetation are well described by the biological spectrum. In the present study, all the three grasslands types experienced similar geographical, climatic, and day length conditions and did not show any significant differences in soil temperature, light intensity or climatic conditions. The dominance of hemicryptophytes and chamaephytes in grassland communities is important in depicting the hemicryptophytic and chamaephytic phytoclimates. In both long forbs and grasses and sedges flowering peaked during the rainy season (September), contrary to short forbs. In short forbs, fruiting and seed maturation peaked in the month of May due to the presence of plants with a short growth cycle. The majority of the forbs started germinating prior to grasses and sedges in order to escape from the competition. In the present study, most of the species initiated flowering during the monsoon season because of the ideal conditions (warm, humid weather, and easy access to water). The seasonal rainfall and soil water availability, together with temperature, have a direct impact on the timing of phenophases in these species. Overall, the findings of the study suggest temperature and precipitation as key determinants of plant phenology, with significant ramifications for the sustainability of ecosystems. Climate change, which is predicted to affect both important environmental factors, i.e., temperature regimes and precipitation patterns, poses a significant threat to ecosystems by disturbing these delicately balanced relationships. The timing of important plant life cycle events like germination, flowering, and senescence will probably change as temperatures increase and precipitation becomes more unpredictable, which might result in decreased plant productivity and biodiversity. The information obtained in this study is crucial in helping policymakers to create policies for the sustainable management and protection of plant resources. For studies in the fields of ecology and the environment, this research provides a baseline of insightful data that will be helpful in comparing and differentiating the vegetation composition in the Himalayas and its ecosystems.

## Figures and Tables

**Table 1:** List of species encountered in different grassland communities.

Scientific Name	Family	Habit	Life Form	Oak Zone	Cypress Zone	Pine Zone	Sources Used for Identification
**TALL FORB**							
*Achyranthes bidentata* Blume	Amaranthaceae	A/P	Th	−	−	+	FON, FN
*Ageratina adenophora* (Spreng.) King & H. Rob.	Asteraceae	P	Ph	+	+	+	FN
*Anaphalis contorta* (D. Don) Hook.f.	Asteraceae	P	Ch	+	+	+	FON, FN
*Artemisia annua* L.	Asteraceae	A	Th	+	+	+	FON
*Bidens pilosa* L.	Asteraceae	A	Th	−	+	+	FN
*Campanula* spp.	Campanulaceae	A/B/P	He	−	−	+	FON, FN
*Cynoglossum furcatum* Wall.	Boraginaceae	A/B	He	+	+	+	FN
*Dicliptera bupleuroides* Nees	Acanthaceae	P	Ch	+	+	+	FON, FN
*Gonostegia hirta* Turcz.	Urticaceae	P	Ge	+	+	+	FON, FN
*Leucas lanata* Benth.	Lamiaceae	P	Ch	+	+	+	FON, FN
*Nepeta leucophylla* Benth.	Lamiaceae	P	Th	−	−	+	FN
*Nepeta racemosa* Lam.	Lamiaceae	P	Th	+	−	−	FON
*Origanum vulgare* L.	Lamiaceae	P	Ch	+	+	+	FON, FN
*Plectranthus japonicus* (Burm.f.) Koidz.	Lamiaceae	P	Ch	+	−	−	FN
*Plectranthus rugosus* Wall. ex Benth.	Lamiaceae	P	Ch	−	+	+	FN
*Polygonum capitatum* Buch.-Ham. ex D. Don.	Polygonaceae	A	Ch	+	−	−	FN
*Polygonum nepalense* Meisn.	Polygonaceae	A	Th	+	−	+	FN
*Reinwardtia indica* Dumort.	Linaceae	P	Ge	+	+	+	FN
*Roscoea purpurea* Sm.	Zingiberaceae	P	Ge	+	−	+	FON, FN
*Senecio nudicaulis* Buch.-Ham. ex D.Don	Asteraceae	P	Ch	+	+	−	FON, FN
*Swertia chirayita* (Roxb.) H. Karst.	Gentinaceae	A	Ch	−	−	+	FN
*Tragopogon gracilis* D. Don	Asteraceae	P/B	Ch	+	−	−	FON, FN
*Verbascum thapsus* L.	Scrophulariaceae	B	He	+	−	+	FON, FN
**GRASSES AND SEDGES**							
*Arthraxon lanceolatus* (Roxb.) Hochst.	Poaceae	P	He	+	+	+	FN
*Arudinella nepalensis* Trin.	Poaceae	P	He	+	+	+	FON, FN
*Chrysopogon serrulatus* Trin.	Poaceae	P	He	+	+	+	FON
*Cymbopogon distans* Nees ex Steud.	Poaceae	P	He	+	+	+	FON, FN
*Cyperus niveus* Retz.	Cyperaceae	P	He	−	−	+	FN
*Cyperus rotundus* L.	Cyperaceae	P	He	+	−	−	FON, FN
*Digitaria cruciata* (Nees) A. Camus	Poaceae	A	He	+	−	+	FON, FN
*Eulalia mollis* (Griseb.) Kuntze	Poaceae	P	He	−	+	−	FON, FN
*Heteropogon contortus* (L.) P. Beauv. ex Roem. & Schult.	Poaceae	P	He	+	+	+	FON
*Koeleria macrantha* (Ledeb.) Schult.	Poaceae	P	He	+	+	−	FN
*Oplismenus compositus* (L.) P. Beauv.	Poaceae	P	He	+	−	+	FON, FN
*Paspalum dilatatum* Poir.	Poaceae	P	He	+	−	−	FON
*Paspalum distichum* L.	Poaceae	P	He	−	+	+	FON
*Saccharum rufipilum* Steud.	Poaceae	P	He	−	+	+	FN
*Sporobolus diander* (Retz.) Beauv.	Poaceae	P	He	+	−	−	FON, FN
*Sporobolus fertilis* (Steud.) W.D.Clayt	Poaceae	P	He	+	−	+	FON
**SHORT FORBS**							
*Arenaria serpyllifolia L.*	Caryophyllaceae	A/P	Ch	−	+	−	FON
*Buchnera hispida* Buch.-Ham. ex D.Don	Orobanchaceae	A	Ch	+	+	+	FON
*Commelina benghalensis* L.	Commelinaceae	A	Ge	+	−	+	FON
*Curculigo orchioides* Gaertn.	Hypoxidaceae	A	Ge	+	−	−	FON
*Cyanotis vaga* D.Don	Commelinaceae	P	Ge	+	+	+	FON
*Desmodium microphyllum* (Thunb.)DC.	Fabaceae	P	He	+	−	+	FON
*Flemingia bracteata* Roxb.	Fabaceae	P	Ge	−	−	+	FN
*Flemingia procumbens* Roxb.	Fabaceae	P	Ge	−	−	+	FON
*Fragaria vesca* L.	Rosaceae	P	Ge	+	+	−	FN
*Galium aparine* L.	Rubiaceae	A	Ch	+	+	−	FON
*Geranium ocellatum* Jacq. ex Cambess.	Geraniaceae	A	He	−	+	+	FN
*Gerbera gossypina* (Royle) Beauverd	Asteraceae	P	Ch	+	−	−	FON
*Himalaiella heteromallla* D. Don	Asteraceae	P	Ch	−	+	+	FON
*Hypoxis aurea* Lour.	Hypoxidaceae	P	Ge	+	−	−	FON
*Justicia diffusa* Willd.	Acanthaceae	P	He	+	+	+	FON
*Micromeria biflora* (Buch.-Ham. ex D.Don) Benth.	Lamiaceae	P	Ge	+	+	+	FON
*Mimosa* spp	Fabaceae	A/P	Ch	+	−	−	FON
*Nicandra physalodes* (L.) Gaertn.	Solanaceae	A	Th	−	−	+	FON
*Oreocome candollei* (Wall. ex DC.)	Apiaceae	P	Th	+	+	−	http://www.flowersofindia.net/
*Oxalis corniculata* L.	Oxalidaceae	A	Ge	+	+	+	FON, FN
*Polygala persicariifolia* DC.	Polygalaceae	A	He	+	−	+	FON, FN
*Salvia lanata* Roxb.	Lamiaceae	P	Ge	−	−	+	FN
*Scutellaria scandens* D. Don	Lamiaceae	P	Ge	+	−	−	FON
*Solanum xanthocarpum Schrad. & Wendl*	Solanaceae	P	Ge	−	−	+	FN
**UNDERSHRUBS**							
*Barleria cristata* L.	Acanthaceae	P	Ch	+	−	+	FN
*Cassia occidentalis* L.	Fabaceae	P	Ge	+	+	−	FN
*Crotolaria alata* Buch. Ham. Ex Roxb.	Fabaceae	A/P	Ge	+	+	+	FN
*Desmodium heterocarpon* (L.) DC.	Fabaceae	P	Ge	+	−	+	FON, FN
*Inula cappa* (Buch.-Ham. ex D.Don) DC.	Asteraceae	P	Ph	−	−	+	FON, FN
*Lepidagathis cuspidata* Nees	Acanthaceae	P	Ge	+	+	+	FN
*Lespedeza eriocarpa* DC.	Papilionaceae	A	Ch	−	+	−	FN

Where A = annual; B = biennial; P = perennial; Ch = chaemaephyte; He = hemicryptophyte; Ge = geophyte; Th = therophyte; Ph = phanerophyte; FON = Flora of Nainital district [34]; FN = Flora Nainitalensis [35]. (‘+’ and ‘−’ signs denotes the presence or absence of a particular species).

**Table 2:** Raunkiaer normal spectrum (%) and biological spectrum (%) of Indian grassland communities.

S. No.	Study Area	Ph	Ch	He	Ge	Th	References
1.	-	46.00	9.00	26.00	6.00	13.00	Raunkiaer [3]
2.	Varanasi	-	3.10	20.30	7.80	68.70	Singh [49]
3.	Varanasi	40.00	6.00	1.00	10.00	43.00	Rao [50]
4.	Kurukshetra	2.09	10.41	18.75	6.25	62.50	Singh and Yadava [47]
5.	Varanasi	-	4.20	19.20	6.30	70.20	Singh and Ambasht [45]
6.	Berhampur	5.70	25.70	14.30	5.70	48.60	Misra and Misra [51]
7.	Berhampur	10.00	26.66	23.33	3.33	36.33	Malana and Misra [52]
8.	Berhampur	5.40	21.60	18.90	2.70	51.30	Rath and Misra [53]
9.	Western Orissa	3.00	21.20	18.20	6.00	51.50	Naik [54]
10.	South Orissa	3.58	17.86	25.00	10.71	42.86	Patnaik [55]
11.	Phulbani	5.71	20.00	11.42	8.57	54.28	Behera and Misra [56]
12.	Bhubaneswar	5.88	29.42	11.76	5.88	47.05	Pradhan [57]
13.	Berhampur	-	25.81	12.90	9.68	51.61	Barik and Misra [58]
14.	Thoubal, Manipur	-	4.84	11.29	14.52	69.35	Devi et al. [59]
15.	Semi-arid grassland, Maharashtra	21.00	17.00	16.00	14.00	32.00	Jyanthi and Jalal [41]
16.	Bangiriposi, Orissa	-	46.88	12.50	6.25	34.37	Rout and Barik [48]
17.	Kumaun Himalaya	2–4.00	20–33.00	31–37.00	28–30.00	5–10.00	Present study

Where Ph = phanerophyte; Ch = chaemaephyte; He = hemicryptophyte; Ge = geophyte; Th = therophyte.

**Table 3:** Description of the study sites.

Forest Zone	Altitude (m)	Latitude (N)	Longitude (E)	Slope (°)	Aspect	Shannon-Weiner Index (H′)	Simpson’s Index (Cd)	Dominant Tree Species	Dominant Shrub Species
Oak	1800–1900	29°22′14′′	79°28′54′′	29.8	South East	1.72	0.21	*Quercus leucotrichophora*	*Arudinaria falcata*
Cypress	1700–1800	29°21′41′′	79°27′45′′	36.2	North East	1.79	0.24	*Cupressus torulosa*	*Berberis asiatica*
Pine	1700–1800	29°23′03′′	79°29′42′′	31.5	South	1.83	0.22	*Pinus roxburghii*	*Randia tetrasperma*

**Table 4:** Soil physico-chemical properties of the study sites.

Zone	Physical Properties of Soil								Chemical Properties of Soil				
	Soil Texture	Gravel (g)	Sand (%)	Silt (%)	Clay (%)	bD (g cm^−3^)	Porosity (%)	WHC (%)	pH	SOC (%)	TN (%)	P (%)	K (%)
Oak	Sandy-clay-loam	65.87	52.15	27.83	20.02	1.31	49.31	62.91	5.9	2.93	0.32	0.0012	0.005
Cypress	Clay-loam	65.49	44.02	25.05	30.92	1.22	52.85	67.75	6.4	2.96	0.26	0.0014	0.007
Pine	Sandy-loam	75.92	55.49	25.97	18.54	1.24	52.10	71.16	5.9	3.67	0.29	0.0016	0.010

Where bD = bulk density; WHC = water holding capacity; SOC = soil organic carbon; TN = total nitrogen; P = available phosphorus; K = available potassium.

## Data Availability

Data are contained within the article.
